# Couples’ Psychological Resources and Marital Satisfaction: The Mediating Role of Marital Support

**DOI:** 10.5964/ejop.11769

**Published:** 2024-11-29

**Authors:** Keren Michael, Hasida Ben-Zur

**Affiliations:** 1 Department of Social Work, Department of Human Services, Max Stern Yezreel Valley College, Yezreel Valley, Israel; 2 School of Social Work, University of Haifa, Haifa, Israel; Dublin City University, Dublin, Ireland

**Keywords:** APIM, couples/dyads, dispositional optimism, marital satisfaction, marital support, psychological resources, sense of mastery

## Abstract

The aim of this study was to assess the interdependent relations among psychological resources, marital support, and marital satisfaction in married couples from the perspective of both dyad members, using the Actor-Partner-Interdependence-Model (APIM) approach. One hundred and fifty-one heterosexual married couples (*N* = 302) completed questionnaires assessing psychological resources (dispositional optimism and sense of mastery), marital support, marital satisfaction, social desirability, and demographic variables. Structural equation modeling (SEM) was used to test a proposed mediation model adopting the dyadic approach. It was found that husbands’ and wives’ own psychological resources contributed positively to their own perceptions of marital support and that marital support mediated the effects of these resources on their own (actor–actor effect) and on their spouses’ marital satisfaction (actor–partner effect). The results highlight the important contribution of each dyad member’s own psychological resources, as well as the valuable role of perceived support as a mediator, regardless of gender. Hence, clinical practice should encourage individuals to invest in maintaining their personal assets and abilities because of their positive effect on expanding intra- and inter- processes of well-being within the marriage.

## Marital Satisfaction and Marital Support

Married people are subjected daily to interspousal interactions that shape their personal and marital well-being. Being married has been shown to be associated with subjective well-being and mental and physical health (Schmitt et al., 2007). Indeed, married people are found to fare better than divorced or widowed persons in terms of well-being as measured by life satisfaction (e.g., Ben-Zur, 2012). While *life satisfaction* refers to the personal aspects of life, *marital satisfaction* is the extent of contentment with different aspects of the marriage (Ostovar et al., 2023). As well as being one of the leading indicators of a good marriage and potentially predicting a long-lasting relationship, it is also a protective factor against adverse outcomes of life events. For example, Omani-Samani et al. (2018) demonstrated negative correlations between marital satisfaction and hospital anxiety or depression among individuals (113 men and 141 women) suffering from couple infertility.

A variety of factors can potentially affect marital satisfaction, including demographics such as the age and gender of each dyad member, personal qualities, and the family’s socioeconomic status (Schmitt et al., 2007). However, in a study by Schmitt et al. (2007), most of the socioeconomic variables and the Big Five personality traits showed non-significant associations with marital satisfaction. Similarly, Omani-Samani et al. (2018) found no association between marital satisfaction and gender, education, or age. Within the family, a variable that can contribute to marital satisfaction is interspousal support.

Similar to social support, *marital support* can take various forms, e.g., tangible, informational, and emotional support. In addition, the amount of support that is received and support adequacy may vary (Lawrence et al., 2008). Marital support may be essential in solving personal and marital problems and in dealing actively with stressful events; marital support can be a buffer between stress and outcomes such as distress, anxiety, and depression. It can be assumed that these functions of interspousal support result in elevated well-being.

In this study, we examined marital satisfaction and its contributing factors among heterosexual couples using the Actor-Partner-Interdependence-Model (APIM) approach (Cook & Kenny, 2005). The APIM approach assesses associations between variables at the dyad member level by estimating the within- and between-partner effects. In this way, individuals’ variables are associated with their own variables (actor effect) but also with those of the variables of the other member (partner effect). This approach is considered the most appropriate to account for dyadic processes (Proyer et al., 2019) because, for example, it perceives husband and wife as a single unit rather than two distinct people and allows the use of paired data (Du et al., 2021). Using the supplement to APIM—the APIMeM (Ledermann et al., 2011)—we tested whether marital support mediates the association between psychological resources considered to be resilience factors and marital satisfaction.

## Resilience and Psychological Resources

*Resilience* in this study context is defined as an attribute characterizing people by the way they overcome stressful circumstances (Connor & Davidson, 2003). When coping effectively with extreme life events or traumatic experiences, the idea of resilience is interlocked well with the concept of resources: “Personal resources are aspects of the self that are generally linked to resiliency” (Hobfoll et al., 2003, p. 632). Conservation of Resources (COR) theory (Hobfoll, 2001) describes resources as those things that people value, obtain, and maintain, and guard against their loss. Examples of resources are objects (house, car), conditions (family, work, health), energies (money, time), and psychological characteristics (dispositional optimism, sense of mastery). Thus, resilience characterizes environments that are rich in personal, material, social, and energy resources; and access to these resources promotes resource growth and prevents resource loss (Hobfoll et al., 2015).

When husbands and wives possess personal resources, such as dispositional optimism, sense of mastery, self-esteem, self-efficacy, or flexibility, they can cope effectively with marital problems and other stressful events, presumably resulting in higher marital satisfaction. Moreover, following Hobfoll et al. (2015), personal resources are expected to strengthen other resources such as marital support, which in turn is likely to contribute to marital satisfaction. Therefore, in this study, we utilized two personal, psychological resources—dispositional optimism and sense of mastery—that play a prominent role in coping effectively with stressors (e.g., Hobfoll, 2001; Lazarus & Folkman, 1984; McEwen et al., 2015).

*Optimism* represents “the extent to which people hold generalized favorable expectancies for their future” (Carver et al., 2010, p. 879). This characteristic leads to continued efforts to deal with problems (Scheier & Carver, 1985) and is linked to task-focused tendencies (Carver et al., 2010). *Mastery* is one of many concepts that are included in the general category of control and represents the perception of a link between self-performed actions and outcomes (Pearlin & Schooler, 1978). This characteristic “refers to the extent to which people see themselves as being in control of the forces that importantly affect their lives” (Pearlin et al., 1981, p. 340). The psychological characteristics of optimism and mastery promote adjustment to stressful events (Gallagher et al., 2019; Julian et al., 2021) and prior empirical research supports this contention. For example, a greater gain of mastery was found to be related to lower depression and anger among low-income women (Hobfoll et al., 2003), and optimism was negatively associated with PTSD among such women (Lillis et al., 2018).

Psychological resources are assumed to contribute not only to adaptation to stressful life events but also to subjective well-being and positive adjustment in everyday life (e.g., Lazarus & Folkman, 1984). Indeed, it was shown that mastery and optimism were positively related to life satisfaction, an indicator of well-being, among both adolescents and adults (Ben-Zur, 2003), among Turkish students and academic staff (Yetim, 2003), and among Israeli Jewish and Arab students (Zeidner & Ben-Zur, 2013). These resources, together with self-esteem and social support, were also shown to mediate the effects of health on students’ general well-being (Ben-Zur, 2020).

The findings described above, and COR theory (Hobfoll, 2001), suggest that optimism and mastery are two personal resources that have the potential to contribute to marital well-being. A tendency to be highly optimistic leads to positive beliefs regarding future occurrences and thus can shape positive views about marital interactions and the ability to solve future intra- marital conflicts. Similarly, since a high level of mastery entails beliefs about one’s ability to control outcomes, it can also lead to the conviction that marital problems and disputes can be overcome.

## Psychological Resources, Marital Satisfaction, and Marital Support

The present study focused on the contributing role of dispositional optimism and sense of mastery to marital well-being through the mediation of marital support. As for the associations between these psychological resources and marital satisfaction, the results obtained thus far are equivocal. Of the two resources, mainly optimism was investigated and was positively related to marital satisfaction in 56 couples in which the woman had breast cancer (Rock et al., 2014) and in 80 couples coping with infertility (Ostovar et al., 2023). In a longitudinal study with couples using APIM approach, for wives but not for their husbands, optimism was found to be related to their own (actor effect) and their husbands’ (partner effect) marital satisfaction (Terveer & Wood, 2014). In contrast, in a study that examined fathers and mothers of children with autism spectrum disorder, also using APIM approach (Ekas et al., 2015), optimism was not significantly correlated with marital satisfaction. Regarding mastery, to the best of our knowledge, no study to date has investigated its effects on marital satisfaction.

As for the associations between psychological resources and marital support, since optimism and mastery are considered resilience resources, it was assumed that such qualities would be expressed in better interpersonal relationships. They can lead to both dyad members’ enhanced ability to acquire marital support, which helps them deal with everyday problems and marital issues by providing advice, performing valuable actions, and/or giving emotional support and sympathetic encouragement. Indeed, some studies that used the APIM approach found positive associations between optimism and marital support (Segel-Karpas & Arbel, 2022; Smith et al., 2013).

Numerous studies showed positive associations between marital support and marital satisfaction (e.g., Işık & Kaya, 2022; Perlowski & Wright, 2021). For example, among 248 married dyads of Israeli male combat commanders and their female spouses (Zitronblat, & Dekel, 2021), higher levels of perceived support by husbands/wives were associated with higher levels of their own marital satisfaction (actor effect), as well as with their spouses’ marital satisfaction (partner effect). Similarly, among 483 young adult married dyads, both husbands and wives reported higher marital satisfaction when wives sensed greater support from their husbands, even if the husbands were drinking heavily (Windle & Windle, 2019). When analyzing various support types, positive associations were reported between four types of interspousal support (emotional, instrumental/information, appraisal, and social companionship) and marital satisfaction for both men and women among 195 married dyads (Yedirir & Hamarta, 2015).

As for the associations between psychological resources, marital support, and marital satisfaction, in an earlier study that used APIM approach, Srivastava et al. (2006) showed that optimism among 108 dyads of dating undergraduates contributed to their perception of support from their boyfriend/girlfriend, and this perceived support mediated the effect of optimism on relationship satisfaction. Regarding mastery, no previous study examined its associations with either marital satisfaction or support.

## Aims and Hypotheses

To sum up, few prior studies have assessed the effects of psychological resources on aspects of marital well-being. It was suggested that the legal and interpersonal commitment is stronger among married couples than among cohabiting couples, who have more joint investments and prescribed social roles (Blekesaune, 2018), and therefore the focus of this study was on married couples. Since it was claimed that marital support leads to marital satisfaction, we tested a SEM model in which psychological resources are associated with marital support, which, in turn, is associated with marital satisfaction among couples. This analysis was examined using APIMeM (the extension of APIM to mediation testing), observing both actor and partner effects. We assumed that one’s own or one’s spouse’s psychological resources of dispositional optimism and sense of mastery affects perceptions of interspousal support, and that this sense of support will lead to one’s own or the spouse’s marital satisfaction.

The study hypotheses were as follows:

- **H1**) There will be positive associations between psychological resources and marital satisfaction among husbands and among wives: a) Individuals’ own psychological resources will be associated with their own marital satisfaction (actor effect). b) Individuals’ own psychological resources will be associated with their spouses’ marital satisfaction (partner effect).
- **H2**) There will be positive associations between psychological resources and marital support among husbands and among wives: a) Individuals’ own resources will be associated with their own marital support (actor effect). b) Individuals’ own resources will be associated with their spouses’ marital support (partner effect).
- **H3**) There will be positive associations between marital support and marital satisfaction among husbands and among wives: a) Individuals’ own marital support will be associated with their own marital satisfaction (actor effect). b) Individuals’ own marital support will be associated with their spouses’ marital satisfaction (partner effect).
- **H4**) Marital support will mediate the associations between psychological resources and marital satisfaction among husbands and among wives: a) One’s own marital support will mediate the association of individuals’ own resources with their own marital satisfaction (actor–actor effect). b) One’s own marital support will mediate the association of individuals’ own resources with their spouses’ marital satisfaction (actor–partner effect). c) One’s own marital support will mediate the association of spouses’ resources with individuals’ own marital satisfaction (partner–actor effect). d) One’s own marital support will mediate the association of spouses’ resources with spouses’ marital satisfaction (partner–partner effect).

## Method

### Sample and Procedure

The sample included 151 heterosexual couples (*N* = 302). This sample size is appropriate according to the Monte Carlo simulation determination for sample size for mediation analysis (APIMeM) within the APIM approach. The calculation revealed that the recommended sample size for the direct effects was between 61 to 111 couples, and for the indirect effects was between 65 to 144 couples (Ledermann et al., 2022).

The couples had been married for an average of 13.59 (*SD* = 11.27) years. The husbands’ mean age was 41.26 (*SD* = 10.14), and they reported an average of 15.09 (*SD* = 2.87) years of education. The wives’ mean age was 38.21 (*SD* = 9.63), and they reported an average of 15.41 (*SD* = 2.38) years of education. Most of the participants were born in Israel (86% of the husbands and 90% of the wives), were Jewish (78% and 79% of husbands and wives, respectively), and about 80% reported having children (1–5).

Graduate students approached married couples in the community within the age range of 30–65 years and who spoke Hebrew fluently. The couples participated in the study voluntarily. Each couple received two assessment packets, and each dyad member completed the questionnaires independently. The respondents were informed that the questionnaires included items related to individual differences in attitudes, feelings, and cognitions, and were assured that their responses would be coded anonymously. The study was approved by the institution’s human subject committee.

### Inventories

Background information included gender, age, number of years of formal education, country of origin, number of years of marriage, and number of children. The correlations between the husbands and the wives’ reports of years of marriage and the number of children were 0.97 and 0.98, respectively, suggesting that their reports in terms of these demographics were accurate. The following questionnaires were used:

#### Dispositional Optimism

Evaluated by a Hebrew version (Ben-Zur, 2012) of the Life Orientation Test (LOT), first introduced by Scheier and Carver (1985). The LOT is composed of eight items (e.g., “*In uncertain times, I usually expect the best*”), rated on a 5-point Likert scale (1 = *Strongly disagree*; 5 = *Strongly agree*). An optimism score is created by computing the mean of all items (after reversing four items) with a high mean score indicating an optimistic tendency. The internal reliability and test-retest of the LOT were satisfactory (α = 0.76, *r_tt_* = 0.79; Scheier & Carver, 1985). In the present study, Cronbach’s alpha was 0.79 for husbands and 0.74 for wives.

#### Sense of Mastery

Assessed using the Hebrew version (Hobfoll & Walfisch, 1984) of a scale developed by Pearlin and Schooler (1978). The scale is composed of seven items (e.g., “*I have little control over the things that happen to me*”) rated on a 7-point Likert scale (1 = *Not at all characteristic of me*; 7 = *Very characteristic of me*). A mean score is computed (after reversing five of the items), with a high score indicating a high level of mastery. Hobfoll and Walfisch (1984) reported test-retest reliability of *r_tt_* = 0.85, with reasonable internal reliability (α = 0.75). In the present study, Cronbach’s alpha was 0.75 both for husbands and for wives.

#### Marital Support

Each one of the dyad members’ perception of his/her spouse’s support was measured by a 6-item Hebrew scale developed by Altus (2004). Each item refers to the ability to turn to the spouse regarding issues such as getting advice, help, or emotional support (e.g., “*I feel I can turn to my spouse when I am hurt by someone*”). The items are rated on a 1–4 Likert scale (1 = *Not at all*; 4 = *Very easily*), with a high score indicating a high level of marital support. Altus (2004) reported internal reliability of α = 0.83 for men. In the present study, Cronbach’s alpha was 0.89 for husbands and 0.86 for wives.

#### Marital Satisfaction

The Hebrew version (Tal-Katz, 2003) of The Kansas Marital Satisfaction Scale (KMSS; Schumm et al., 1985) was used to measure husbands and wives’ marital satisfaction. The scale consists of three items referring to marital satisfaction (e.g., “*How satisfied are you with your husband/wife as a spouse?*”) and is rated on a 1–7 rating scale (1 = *Not satisfied at all*; 7 = *Very satisfied*). The scale’s reliability and validity were recently reconfirmed (Omani-Samani et al., 2018) with high reliability values for both men and women (α = 0.91 and .89, respectively). In the present study, Cronbach’s alpha was 0.95 for husbands and 0.96 for wives.

#### Social Desirability

A Hebrew adaptation (Ben-Zur & Michael, 2020) of the 8-item Social Desirability Questionnaire (Crowne & Marlowe, 1964) was employed to control for potential social desirability in responding to the self-report measures. Each item is marked as true or untrue, and the scale score is the total count of answers denoting high social desirability (α = 0.67; Ben-Zur & Michael, 2020*).* In the present study, the Kuder-Richardson (KR-20) coefficient was 0.70 for husbands and 0.64 for wives.

### Data Analysis

Data analyses were conducted by the SPSS program (Version 25). R software via Monte Carlo simulation [packages: “lavaan” (Rosseel, 2012), “paramtest” (Hughes, 2022), “simsen” (Pornprasertmanit et al., 2022)] was used for the determination of the sample size (Ledermann et al., 2022). Means and standard deviations were carried out, and paired samples *t*-tests were performed to examine the differences between the husbands’ and wives’ variables. Pearson correlations among dispositional optimism, sense of mastery, marital support, marital satisfaction, and social desirability were tested separately for husbands and wives (actor-level correlations) and between the spouses’ variables (partner-level correlations). R software via “lavaan” package (Rosseel, 2012) was used for CFA and APIMeM. CFA via the ML estimation method was performed on the item scores of dispositional optimism and sense of mastery to examine the distribution to two factors. APIMeM using SEM via the MLR estimation method was used to test the proposed research model, in which, initially, psychological resources were tested as associated with marital satisfaction (H1; Path c). Then, psychological resources were tested as associated with marital support (H2; Path a); which, in turn, was tested as associated with marital satisfaction (H3; Path b), and finally, marital support was tested as a mediator of the associations between psychological resources and marital satisfaction (H4; Path ab).

Within the proposed research model, according to Ackermann et al. (2011), the distensibility of parameter estimates between husbands and wife was tested through three steps: initially, examining similarities of the associations among husbands and wives (B scores); then, examining similarities of the means in the variables psychological resources, marital support, and marital satisfaction; finally, examining similarities of the variance in theses variables among husbands and wives.

Hence, the proposed research model included some restrictions: The association between the same variable for the different actors (husbands and wives) was equal (for example, the association between husbands’ marital support and husbands’ marital satisfaction was equal to the association between wives’ marital support and wives’ marital satisfaction). Additionally, all the associations between same variable for different partners were equal (for example, the association between husbands’ marital support and wives’ marital satisfaction was equal to the association between wives’ marital support and husbands’ marital satisfaction). Lastly, all the variances of the same variables for different partners were equal (for example, the variance of marital satisfaction for husbands were equal to the variance of marital satisfaction for wives). However, the means of the same variables for different partners were not equal. In the model tested, all combinations of actor/partner effects were tested: actor, partner, actor–actor, actor–partner, partner–actor, and partner–partner.

APIMeM using SEM was also performed to test an alternative option: the above model but with no restrictions. Another alternative option included controlling demographic variables in the model. For this examination, correlations were initially calculated between marital support/marital satisfaction and the demographic variables. Another alternative option included performing APIM using SEM to test a model, in which dispositional optimism, mastery, and marital support were three indicators of the latent variable of psychological resources, which was tested as associated with marital satisfaction. When comparing between the proposed research model and alternative models, Chi-square tests for goodness of fit were calculated.

Additionally, all models’ fit indices were evaluated based on Hair et al. (2010) and on Hu and Bentler’s (1999) recommendations for acceptable threshold value levels: ratio of Chi-square to degrees of freedom (χ^2^/df) less than 5.00; Tucker-Lewis index (TLI) and comparative fit index (CFI) greater than 0.90; root mean square error of approximation (RMSEA) and standardized root mean square residual (SRMR) less than 0.08. Bootstrapping procedure (*n* = 5,000) and 95% (CIs) was used for testing the indirect effects. In general, the defined significance level was set to 5% (*p* < .05).

## Results

### Preliminary Analysis

A preliminary analysis was conducted to examine differences between husbands and wives regarding the study variables, as well as correlations among the variables. Table 1 presents participants’ means and standard deviations as observed in the study and the differences between husbands and wives in the variables, using paired samples *t*-tests.

**Table 1:** Differences in the Study Variables Between Husbands and Wives

	Husbands		Wives		
Variable	*Mean*	*SD*	*Mean*	*SD*	*t(df)*
Dispositional optimism	3.70	0.70	3.70	0.64	-0.18(150)
Sense of mastery	5.13	1.04	5.03	0.99	1.07(150)
Marital support	3.43	0.60	3.57	0.49	-2.62(150)**
Marital satisfaction	6.14	1.09	6.03	1.09	1.47(150)
Social desirability	1.55	0.28	1.60	0.26	-0.45(150)

*p < .05. **p < .01. ***p < .001.

As seen in Table 1, husbands and wives did not differ regarding levels of dispositional optimism, sense of mastery, marital satisfaction, or social desirability, except for wives’ perceived marital support, which was higher than that of their husbands.

Table 2 presents the Pearson correlations within the husbands’ and the wives’ variables separately and between the dyad members’ variables.

**Table 2:** Pearson Correlations Among the Study Variables

Variable	2	3	4	5	6	7	8	9	10
1.Dispositional optimism H	0.49***	0.33***	0.25**	0.15	0.26***	0.21*	0.20*	0.24**	0.08
2.Sense of mastery H		0.26***	0.23**	0.15	0.18*	0.37***	0.28***	0.28***	-0.00
3.Marital support H			0.68***	0.11	0.21**	0.32***	0.34***	0.40***	0.09
4.Marital satisfaction H				0.07	0.27***	0.25**	0.42***	0.63***	0.07
5.Social desirability H					-0.05	0.03	-0.04	0.00	0.30***
6.Dispositional optimism W						0.55***	0.35***	0.25**	0.30***
7.Sense of mastery W							0.27***	0.19*	0.17*
8.Marital support W								0.62***	0.05
9.Marital satisfaction W									0.16*
10.Social desirability W									–

*Note*. H = husbands; W = wives.

*p < .05. **p < .01. ***p < .001.

As seen in Table 2, in each gender, dispositional optimism, sense of mastery, marital support, and marital satisfaction were positively inter-correlated. Additionally, husbands’ dispositional optimism, sense of mastery, marital support, and marital satisfaction were positively inter-correlated with their wives’ variables. As for social desirability, only among wives, was it positively correlated with dispositional optimism, sense of mastery, and marital satisfaction. Finally, husbands’ social desirability was positively correlated with wives’ social desirability.

To sum up Table 2 according to Cohen’s (1988) interpretation of the strength in Pearson’s bivariate correlations, we can see that the correlations between dispositional optimism and sense of mastery, as well as the correlations between marital support and marital satisfaction for each gender were strong (i.e., large effect size occurs when *r* value is more than .5). Similarly, the correlation between husbands’ marital satisfaction and wives’ marital satisfaction was also strong.

### APIMeM Model Testing Whether Marital Support Mediates the Associations Between Psychological Resources and Marital Satisfaction

Initially, CFA was run on the item scores of dispositional optimism and sense of mastery among husbands and among wives, to test whether the questionnaires are divided into two separated scales. The model, presented in Table 3 as Model 1, yielded worse fit indices: χ^2^ = 908.870, *df* = 399, *p* < .001; χ^2^/*df* = 2.28; TLI = 0.660; CFI = .688; RMSEA [90% CI] = .092 [0.084, 0.100]; SRMR = 0.120. To improve the model, we used parcel method (Little at al., 2013) by aggregating two or three items. As seen in Table 3, Model 2 had good model fit: χ^2^ = 93.953, *df* = 71, *p* = .036; χ^2^/*df* = 1.32; TLI = 0.964; CFI = .972; RMSEA [90% CI] = .046 [0.013, 0.070]; SRMR = 0.054. This model revealed the two expected separated scales: dispositional optimism and sense of mastery for each population.

**Table 3:** Factor Loadings for Each Item of Dispositional Optimism and Sense of Mastery for Husbands and Wives, for Model 1 and Model 2

	Standardized Factor Loadings			
	Husbands		Wives	
Items Description	Model 1	Model 2	Model 1	Model 2
Dispositional optimism
I always look on the bright side of things	0.90	0.74	0.90	0.69
If something can go wrong for me, it will	0.23		0.06	
I’m always optimistic about my future	0.87	0.75	0.95	0.72
I hardly ever expect things to go my way	0.26		0.12	
In uncertain times, I usually expect the best	0.70	0.84	0.65	0.74
Things never work out the way I want them to	0.31		0.20	
I’m a believer in the idea that “every cloud has a silver lining”	0.54	0.67	0.55	0.76
I rarely count on good things happening to me	0.37		0.38	
Sense of mastery				
There is really no way I can solve some of the problems I have	0.73	0.79	0.75	0.73
I often feel helpless in dealing with the problems of life	0.41		0.41	
There is little I can do to change many of the important things in my life	0.72	0.88	0.79	0.74
I can do just about anything I really set my mind to do	0.48		0.41	
Sometimes I feel that I’m being pushed around in life	0.59	0.62	0.52	0.67
I have little control over the things happen to me	0.48		0.66	
What happens to me in the future mostly depends on me	0.56		0.30	

Based on the CFA analysis and the positive correlations between the optimism and mastery factors (for both husbands and wives: *r* = .57 and .67, respectively), these two variables were used as indicators of the latent variables of psychological resources within the proposed research model.

Hence, in the proposed research model, the latent psychological resources variables were the predictors, marital support variables were the mediators, and marital satisfaction variables were the predicted. The model includes the various combinations of actor/partner effects: actor, partner, actor–actor, actor–partner, partner–actor, and partner–partner. A preliminary analysis that compared two models, with and without restrictions, in order to choose the most parsimonious model, showed that the difference between the models were not significant (χ^2^ = 9.62, *df* = 12, *p* = .650). Thus, we preferred the one with the restrictions.

The model generated very good fit indices: χ^2^ = 34.407, *df* = 21, *p* = .033; χ^2^/*df* = 1.64; TLI = 0.955; CFI = .966; RMSEA [90% CI] = .066 [0.019, 0.104]; SRMR = 0.056.

**Figure 1:**
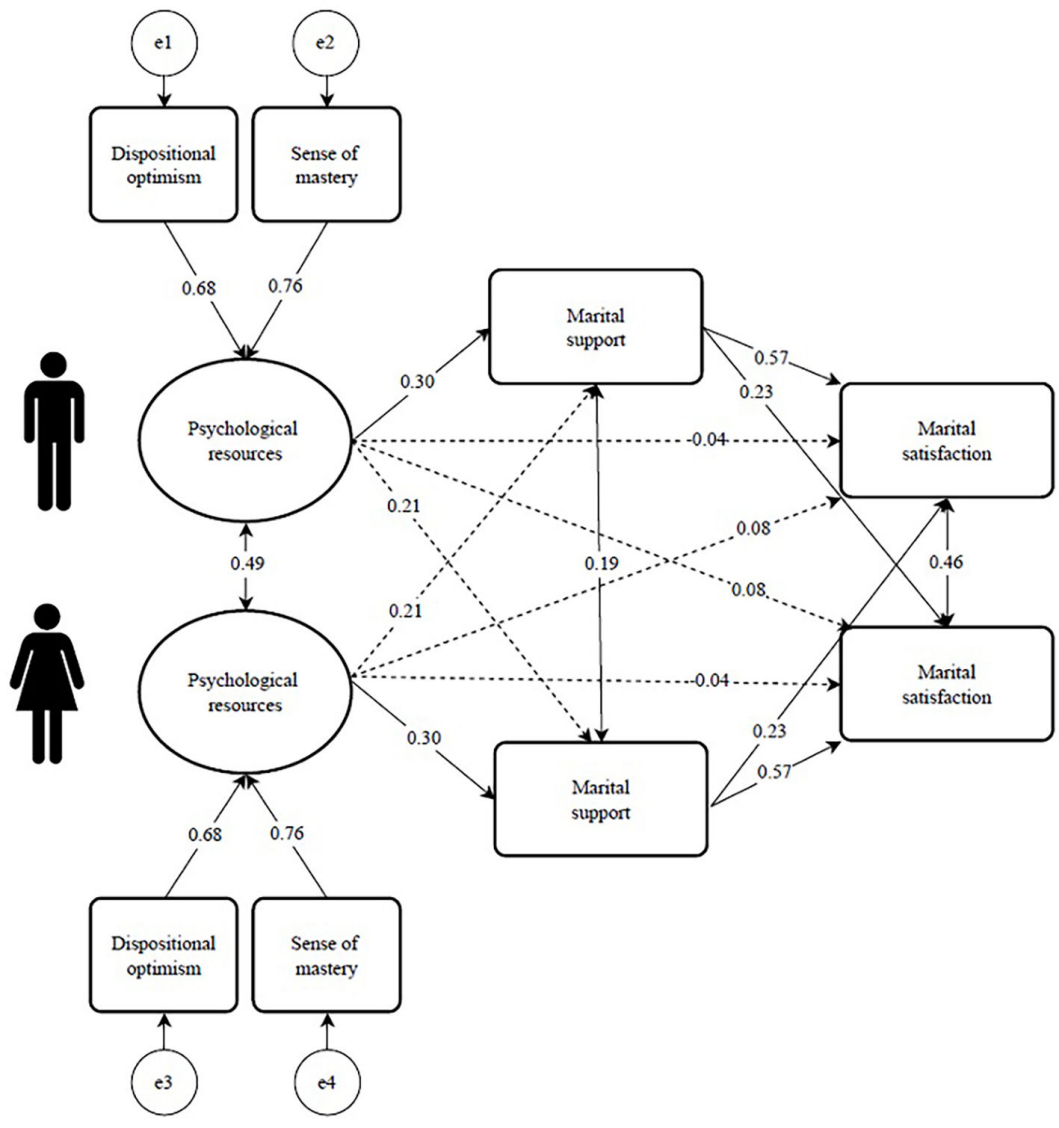
Empirical SEM APIMeM Model for Marital Support Mediating the Associations Between Psychological Resources and Marital Satisfaction of Husbands and Wives *Note*. Solid-line arrow represents significant results. Dashed-line arrow represents insignificant results. In the associations between psychological resources and marital satisfaction, the figure presents c’ (rather than the initial state of c).

As seen in Table 4 (expressed in B scores) and Figure 1 (expressed in beta scores), husbands’ and wives’ psychological resources were associated with their own and their spouses’ marital support, supporting H1a (actor effect) and H1b (partner effect) for the total effect (i.e., Path c). However, the direct effect (i.e., Path c’) of these associations was not significant. Additionally, the resources of both husbands and wives were associated with their own marital support, supporting H2a (actor effect), but not with their spouses’ support, contradicting H2b (partner effect). Additionally, husbands’ and wives’ own marital support was highly associated with their own marital satisfaction, supporting H3a (actor effect), and to a lesser degree, but still significant, with their spouses’ marital satisfaction, supporting H3b (partner effect).

**Table 4:** Total, Direct, and Indirect Effects of Psychological Resources on Marital Satisfaction Through Marital Support

Paths Description	B	SE	90% CI [LL,UL]
Psychological resources-> Marital satisfaction (A)—Path c	0.18	0.08	0.015, 0.337
Psychological resources-> Marital satisfaction (P)—Path c	0.27	0.07	0.132, 0.416
Psychological resources-> Marital satisfaction (A)—Path c’	-0.05	0.06	-0.163, 0.073
Psychological resources-> Marital satisfaction (P)—Path c’	0.08	0.07	-0.049, 0.215
Psychological resources-> Marital support (A)—Path a	0.16	0.07	0.031, 0.289
Psychological resources-> Marital support (P)—Path a	0.11	0.06	-0.002, 0.227
Marital support-> Marital satisfaction (A)—Path b	1.07	0.11	0.849, 1.297
Marital support-> Marital satisfaction (P)—Path b	0.44	0.10	0.252, 0.625
Psychological resources-> Marital support-> Marital satisfaction (A–A)—Path ab	0.17	0.08	0.024, 0.320
Psychological resources-> Marital support-> Marital satisfaction (A–P)—Path ab	0.07	0.03	0.013, 0.127
Psychological resources-> Marital support-> Marital satisfaction (P–A)—Path ab	0.12	0.06	-0.003, 0.244
Psychological resources-> Marital support-> Marital satisfaction (P–P)—Path ab	0.05	0.03	-0.010, 0.109

*Note*. A = actor; P = partner.

Finally, regarding the indirect effects, husbands’ and wives’ own marital support mediated the associations between their own resources and their own marital satisfaction, supporting H4a (actor–actor effect). Similarly, husbands’ and wives’ own marital support mediated the associations between their own resources and their spouses’ marital satisfaction, supporting H4b (actor–partner effect). However, husbands’ and wives’ own marital support did not mediate the associations between their spouses’ resources and their own marital satisfaction, contradicting H4c (partner–actor effect). Similarly, husbands’ and wives’ own marital support did not mediate the associations between their spouses’ resources and their spouses’ marital satisfaction, contradicting H4d (partner–partner effect).

We also examined alternative models, besides the proposed research model, to verify the best model for analyzing the data. One possible model, not including mediation, is one in which dispositional optimism, mastery, and marital support are three indicators of the latent variable of resources. This alternative model leans on the theoretical assumption of Hobfoll et al. (2003) that marital support is correlated with psychological resources and can be considered an intimate resource. Results showed that the fit indices of this alternative model were not acceptable: χ^2^ = 184.62, *df* = 25, *p* < .001; χ^2^/*df* = 7.38; TLI = 0.553; CFI = .601; RMSEA = .208 (CI = 0.180, 0.236); SRMR = 0.177. In the alternative model, the Chi-square and the AIC values were higher than in the proposed research model. Hence, this alternative model is a worse one.

Additionally, in order to examine the need for controlling demographic variables in the model, the correlation between age, years of education, SES, level of religiously, and the mediator (marital support) were performed among each gender, as well as between these demographic variables and the outcome variable (marital satisfaction). The results of most of these correlations were insignificant, and those who were significant had a weak effect size (Cohen, 1988). Hence, trying various models with the participants’ background data was unnecessary.

## Discussion

The aim of the present study was to assess the contribution of the psychological resources of dispositional optimism and sense of mastery, which are considered resilience factors, to marital satisfaction, through the mediation of marital support. The study results showed that the psychological resources of both husbands and wives were associated with their own and their spouses’ marital satisfaction, supporting H1 for both actor and partner effects. Additionally, the psychological resources of both husbands and wives were associated with their own marital support, supporting H2 for the actor effect. Furthermore, marital support of both dyad members was associated with their own and their spouses’ marital satisfaction, supporting H3 for both actor and partner effects. Finally, marital support mediated the associations between psychological resources and marital satisfaction of both husbands and wives, when the resources were their own. In that way, the actor–actor effect and the actor–partner effect were supported (H4a, H4b, respectively). However, when the resources were their spouses’, marital support did not serve as a mediator.

The most prominent general meaning of the findings relates to the contribution of psychological resources to marital well-being, as assessed by marital support and marital satisfaction. Previous research assessed the ameliorating effects of these resources mainly in the context of stress and trauma (e.g., Ben-Zur & Michael, 2012; Hobfoll et al., 2003; Lillis et al., 2018), and some studies also showed their effects on personal well-being. For example, optimism was found to be positively related to various positive outcomes in life (Terveer & Wood, 2014), such as subjective well-being, health, and success (Forgeard & Seligman, 2012). Mastery was found to be associated with better mental and physical quality of life among people with multiple sclerosis (O’Kearney et al., 2020). However, only one study with very young dating undergraduates showed significant optimism effects on perceived boyfriend/girlfriend support and relationship satisfaction (Srivastava et al., 2006), while Ekas et al. (2015) found no optimism effects on marital processes. Several other studies reported effects of optimism on marital satisfaction (e.g., Ostovar et al., 2023; Rock et al., 2014), and no study, to our knowledge, showed the effects of mastery on marital processes.

The present study, conducted among couples with a wide range of years of marriage, showed that the personal resilience factors of dispositional optimism and sense of mastery can be related to perceiving one’s spouse as supportive in a variety of areas such as giving advice, actual help, and emotional support, and that support was associated with marital satisfaction. This finding means that people with high optimism and mastery may possess an enhanced ability to gain more support from their spouses. Additionally, when individuals expect good things to happen in the future and are in control of their present, they can also see others—especially their loved ones—in a positive manner. These perceptions may assist them to perceive their spouses as supportive and helpful. In contrast, a lack of personal resources may create situations where, despite the spouses’ attempts to be there for them or to provide them with actual help, these efforts may not be interpreted as support.

Furthermore, it should be noted that marital support, as discussed above, is also considered a resource (intimate resource; Hobfoll et al., 2003), and family support is measured as one of the components of the resource of social support (Zimet et al., 1988). The findings showing that optimism and mastery are associated with marital support are also in line with the COR theory regarding resource gains and losses (Hobfoll et al., 2015), which suggests that individuals will be endowed with an array of resources. In other words, rather than replacing each other, resources either accumulate or are reduced as an array.

One’s own marital support mediated the effects of individuals’ own resources on their own marital satisfaction (actor–actor effect), but more interestingly, also on their spouses’ marital satisfaction (actor–partner effect). These findings were significant for both dyad members, suggesting that resilience, as exemplified by individuals’ personal psychological resources, and the perception of support are essential for the marital satisfaction of both members of the couple. In that sense, clinical practice should encourage individuals to invest more in maintaining their personal assets and abilities because of their positive effect on expanding intra- and inter-processes of well-being within the marriage. These personal resilience qualities cannot be efficiently utilized without people’s valuable belief that they are receiving emotional, instrumental, cognitive, or other feedback from their mates, as seen in the mediating role of marital support.

Additionally, the results emphasized that leaning on the spouses’ abilities and resources is not enough. In that way, the actor effect in the association between the resources and the marital support was significant, but not the partner effect. These findings led to the different mediation paths, demonstrating that one’s own resources are the more robust anchor in marital support. Thus, individuals’ own resources contribute to the well-being of both dyad members through their own perceived support. It should be noted that the qualities and processes found in this study are important for both men and women, since no different paths were observed among husbands and among wives. Hence, we can see that, regardless of gender, psychological resources are very dominant in coping and well-being. Such reciprocal relationships that include both resources and perceived support can contribute to a long-lasting, happy marriage and have implications for family treatments that deal with dissatisfaction and conflicts in interspousal relationships.

### Strengths and Limitations

The main advantage of the study was the use of a sample of dyads, which provided two sources of reports from both dyad members, allowing the use of interspousal correlations and the APIM approach. Additionally, social desirability was measured for both members of the couple; it did not affect the personal and interspousal correlations, thus lending some validity to this mode of measurement.

The main disadvantage was the cross-sectional nature of the study, which does not allow a firm establishment of cause-and-effect directions. We tested a research model according to which marital support mediated the associations between psychological resources and marital satisfaction, and this was confirmed by SEM analysis that provided very good fit indices for this model, and better indices than those provided for alternative models.

Future studies may continue the investigation using longitudinal designs and intervention studies that can shed more light on the complex relations between psychological resources and marital processes. Studies such as these can contribute to the clinical treatment of marital problems and bolster couples’ well-being.
